# 4-*tert*-Butyl­pyridinium chloride–4,4′-(propane-2,2-di­yl)bis­(2,6-di­methyl­phenol)–toluene (2/2/1)

**DOI:** 10.1107/S1600536814004942

**Published:** 2014-03-12

**Authors:** Alastair J. Nielson, Joyce M. Waters

**Affiliations:** a Chemistry, Institute of Natural and Mathematical Sciences, Massey University at Albany, PO Box 102904, North Shore Mail Centre, Auckland, New Zealand

## Abstract

In the title solvated salt, C_9_H_14_N^+^·Cl^−^·C_19_H_24_O_2_·0.5C_7_H_7_, two mol­ecules of 4,4′-(propane-2,2-di­yl)bis­(2,6-di­methyl­phenol) are linked *via* O—H⋯Cl hydrogen bonds to two chloride ions, each of which is also engaged in N—H⋯Cl hydrogen bonding to a 4-*tert*-butyl­pyridinium cation, giving a cyclic hydrogen-bonded entity centred at 1/2, 1/2, 1/2. The toluene solvent mol­ecule resides in the lattice and resides on an inversion centre; the disorder of the methyl group requires it to have a site-occupancy factor of 0.5. No crystal packing channels are observed.

## Related literature   

For general background to hydrogen-bond structural information, see: Hamilton & Ibers (1968 ▶). For hydrogen bonding between phenols and nitro­gen bases, see: Coupar *et al.* (1997 ▶); Steiner *et al.* (2000 ▶). For hydrogen bonding in phenol mol­ecules, see: Prout *et al.* (1988 ▶); Ziemer & Surygina, (2000 ▶). For the structure of a related *bis*-phenol mol­ecule, see: Okada (1996 ▶). For hydrogen bonds between pyridinium hydro­chloride and OH-containing mol­ecules, see: Sykora & Cioffi (2007 ▶); Hossain *et al.* (1988 ▶). For structural data pertaining to pyridinium hydro­halides, see: Faber *et al.* (1999 ▶); Hensen *et al.* (1988 ▶); Mootz & Hocken (1989 ▶); van de Streek *et al.* (2010 ▶).
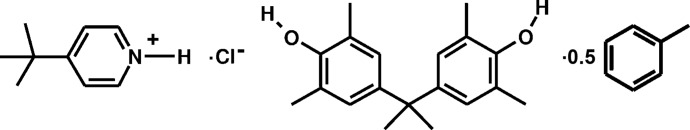


## Experimental   

### 

#### Crystal data  

C_9_H_14_N^+^·Cl^−^·C_19_H_24_O_2_·0.5C_7_H_7_
*M* *_r_* = 501.60
Monoclinic, 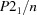

*a* = 13.5656 (9) Å
*b* = 14.3215 (9) Å
*c* = 15.787 (1) Å
β = 112.186 (1)°
*V* = 2840.0 (3) Å^3^
*Z* = 4
Mo *K*α radiation
μ = 0.16 mm^−1^
*T* = 150 K
0.39 × 0.28 × 0.02 mm

#### Data collection  

Siemens SMART diffractometer
Absorption correction: multi-scan (Blessing, 1995 ▶) *T*_min_ = 0.700, *T*_max_ = 0.821
14535 measured reflections
4989 independent reflections
3253 reflections with *I* > 2σ(*I*)
*R*_int_ = 0.053

#### Refinement  

*R*[*F* ^2^ > 2σ(*F* ^2^)] = 0.052
*wR*(*F* ^2^) = 0.114
*S* = 1.08
4989 reflections
379 parameters
H atoms treated by a mixture of independent and constrained refinement
Δρ_max_ = 0.22 e Å^−3^
Δρ_min_ = −0.20 e Å^−3^

### 

Data collection: *SMART* (Siemens, 1995 ▶); cell refinement: *SAINT* (Siemens, 1995 ▶); data reduction: *SAINT*; program(s) used to solve structure: *SHELXS97* (Sheldrick, 2008 ▶); program(s) used to refine structure: *SHELXL97* (Sheldrick, 2008 ▶); molecular graphics: *ORTEP-3 for Windows* (Farrugia, 2012 ▶); software used to prepare material for publication: *SHELXL97*.

## Supplementary Material

Crystal structure: contains datablock(s) I, global. DOI: 10.1107/S1600536814004942/gg2132sup1.cif

Structure factors: contains datablock(s) I. DOI: 10.1107/S1600536814004942/gg2132Isup2.hkl

CCDC reference: 989768

Additional supporting information:  crystallographic information; 3D view; checkCIF report

## Figures and Tables

**Table 1:** Hydrogen-bond geometry (Å, °)

*D*—H⋯*A*	*D*—H	H⋯*A*	*D*⋯*A*	*D*—H⋯*A*
N20—H20⋯Cl	1.06 (4)	1.98 (4)	3.035 (3)	173 (3)
O2—H2⋯Cl^i^	0.85 (3)	2.30 (3)	3.079 (2)	153 (3)
O1—H1⋯Cl	0.90 (4)	2.27 (4)	3.118 (2)	157 (3)

Symmetry code: (i) 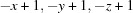.

**Fig. 1.**
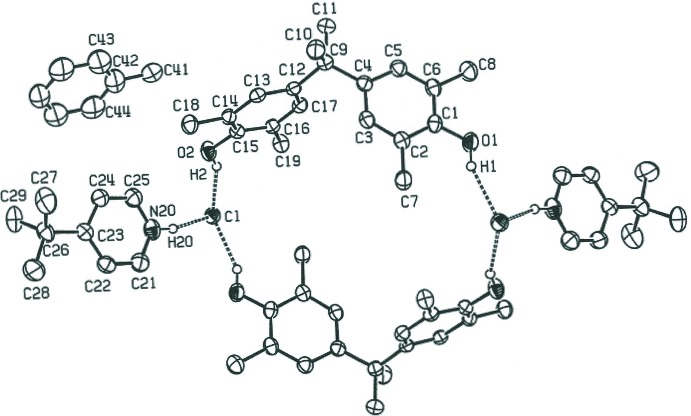
ORTEP diagram of the title compound showing the numbering system and the H-bonding network. H not involved in this bonding have been omitted for reasons of clarity. Note also that only one of the disordered toluene methyl substituents has been included. Thermal ellipsoids have been drawn at the 50% probability level.

**Fig. 2.**
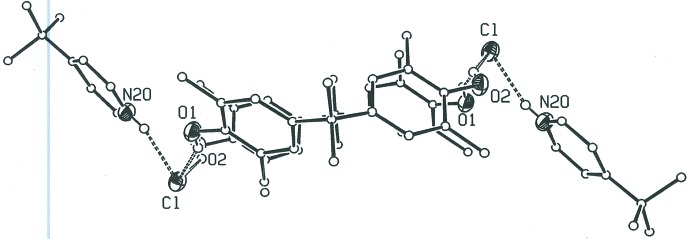
Edge-on ORTEP diagram showing the pyramidal structure about the Cl ions

## supplementary crystallographic information

### 2.1. Synthesis and crystallization

The title compound crystallized from toluene as very thin colourless plates from the reaction between TiCl_4_ and 4,4'-(propane-2,2-diyl)bis­(2,6-di­methyl­phenol) in the presence of 4-*tert*-butyl­pyridine.

### 2.2. Refinement

Crystal data, data collection and structure refinement details are summarized in Table 1. All H atoms (except H1 and H2) were placed in calculated positions and in the refinement allowed to ride on the C atoms to which they were attached. H1 and H2 were located from a difference map and their positions were then allowed to ride on their oxygen atoms. All H atoms were assigned fixed isotropic thermal parameters. Disorder was associated with *tert*-butyl substituent on the pyridinium ion and two alternative positions were found for this group with site occupancy factors of 0.62 and 0.38. Both were refined as a rigid group. A half-weighted molecule of toluene disordered across a centre of symmetry was identified in the asymmetric unit. The numbering system used for the diphenol and pyridinium moieties is indicated in figure 1, where the linking of the moities *via* the chloride ion is shown.

### 3. Results and discussion

As part of a study into the reactions of TiCl_4_ with various phenols in the presence of pyridines, crystals of the title compound were isolated from a toluene solution. X-ray structural analysis revealed two molecules of (CH_3_)_2_C(C_6_H_2_Me_2_OH)_2_ linked together by hydrogen bonds to 2 chloride ions which in turn are further linked to two 4-*tert*-butyl pyridinium-H ions about a centre of symmetry to form a ring of atoms (Figure 1). No Ti atom was present. Distances of 3.118 (2) and 3.079 (2) Å were observed for Cl···O1 and Cl···O2 respectively. The H contact distances and O–H···Cl angles observed were 2.27 (4) Å, 157 (3) ° and 2.30 (3) Å, 153 (3) ° for H1 and H2, respectively. The Cl is also H-bound to H20 of the pyridinium-H ion with Cl···N20 and Cl···H20 approaches of 3.035 (3) and 1.98 (4) Å respectively. The angle N20–H20···Cl is 173 (3) °. These distances are typical of such H-bonded distances as described elsewhere (Hamilton & Ibers, 1968). H-bonds between N bases and phenolic moieties are not uncommon (Coupar *et al.* 1997) and the inter­action is often strong (Steiner *et al.* 2000). However a Cl ion linking a phenol and an ammine hydro­cloride as occurs in the title compound has apparently not been observed before. A single link between pyridinium hydro­chloride and the OH hydrogen of tri­phenyl­methanol has been observed (Sykora & Cioffi, 2007) as has a similar link with the OH group of 1,1,3,3-tetra­phenyl-1,3-disiloxanediol (Hossain *et al.* 1988).

The N20 to Cl separation for the 4-*tert*-butyl­pyridinium hydro­chloride in the present compound [3.035 (3) Å] compares with that found for 4-methyl­pyridinium hydro­chloride itself at 2.999 (2) Å, 2.981 (4) Å in 3-methyl­pyridinium hydro­chloride and 3.162 (2) Å in 4-methyl­pyridinium hydro­bromide (Faber *et al.* 1999). Taking into account the imprecise positioning of hydrogen atoms in X-ray crystal structures, in the latter three compounds the N—H···Cl bond angles are 180 °, 177 (5)° [and 165 (5) °] and 173 (3) ° whereas in the present compound this angle is 173 (3) °. For (C_6_H_5_)_3_COH·C_5_H_6_N^+^·Cl^-^ the N to Cl separation is 3.008 (2) Å and the N—H···Cl bond angle is 169 ° (Sykora & Cioffi, 2007). It is noted here that for pyridinium hydro­chloride itself two polymorphs found by crystallography (Hensen *et al.* 1988; Mootz & Hocken, 1989) have been substanti­ated by dispersion-corrected density functional theory calculations (van de Streek *et al.* 2010).

The O to Cl separations in the title compound are 3.079 (2) and 3.118 (2) Å and these compare with 3.134 (1) Å in (C_6_H_5_)_3_COH·C_5_H_6_N^+^·Cl^-^ (Sykora & Cioffi, 2007). The C1–O1 bond length in the present compound [1.382 (3) Å] is not significantly different from the C15–O2 bond length [1.378 (3) Å] at the opposite end of the bis-phenol. In comparison, 2,4,6-tri­methyl­phenol which is structurally similar to the phenolic portion of the present molecule but contains a hydrogen bonded O—H···OH system, has a C–O bond length of 1.386 (2) Å (Ziemer & Surygina, 2000). 2,6-Diiso­propyl­phenol which also has a hydrogen bonded O—H···OH system has C–O bond lengths of 1.480 (7) Å (C—O—H···O section) and 1.382 (8) Å (C—O···H section) (Prout *et al.* 1988).

The chlorines in the present lattice form a pyramidal structure in the inter­actions with their 3 H-bonded neighbours (Figure 2). Angles are 118, 79 and 69 ° for O1···Cl···O2, O1···Cl···N20 and O2···Cl···N20 respectively and 117, 82 and 76 ° for H1···Cl···H2, H1···Cl···H20 and H2···Cl···H20 respectively. Within the phenolic moiety and the pyrdinium-H ion bond distances and angles are unremarkable. The two phenyl rings of the former (C1—C6 and C12—C17) are inclined to one another at an angle of 84.78 (8) °, a value which is similar to the values reported [86.9 (2), 83.6 (2) and 79.2 (2) °] for the three independent molecules in the asymmetric unit of (CH_3_)_2_C(C_6_H_4_OH)_2_ (*bis*phenol A) (Okada, 1996). Two disordered sites were found for the terminal tertiary butyl substitiuent on the pyridinium-H group. Toluene solvent molecules occupy space between the H-bound units in the crystal lying across centres of symmetry. No close approaches of this solvent to the other molecules are observed. The crystal packing shows that no channels are developed in the overall structure.

### Crystal data

**Table:**

C_9_H_14_N^+^·Cl^−^·C_19_H_24_O_2_·0.5C_7_H_7_	*F*(000) = 1082
*M**_r_* = 501.60	*D*_x_ = 1.173 Mg m^−^^3^
Monoclinic, *P*2_1_/*n*	Mo *K*α radiation, λ = 0.71073 Å
Hall symbol: -P 2yn	Cell parameters from 7233 reflections
*a* = 13.5656 (9) Å	θ = 2.0–25.1°
*b* = 14.3215 (9) Å	µ = 0.16 mm^−^^1^
*c* = 15.787 (1) Å	*T* = 150 K
β = 112.186 (1)°	Thin plate, colourless
*V* = 2840.0 (3) Å^3^	0.39 × 0.28 × 0.02 mm
*Z* = 4	

### Data collection

**Table:**

Siemens SMART diffractometer	4989 independent reflections
Radiation source: fine-focus sealed tube	3253 reflections with *I* > 2σ(*I*)
Graphite monochromator	*R*_int_ = 0.053
area–detector ω scan	θ_max_ = 25.1°, θ_min_ = 2.0°
Absorption correction: multi-scan (Blessing, 1995)	*h* = −16→10
*T*_min_ = 0.700, *T*_max_ = 0.821	*k* = −17→15
14535 measured reflections	*l* = −18→18

### Refinement

**Table:**

Refinement on *F*^2^	Secondary atom site location: difference Fourier map
Least-squares matrix: full	Hydrogen site location: inferred from neighbouring sites
*R*[*F*^2^ > 2σ(*F*^2^)] = 0.052	H atoms treated by a mixture of independent and constrained refinement
*wR*(*F*^2^) = 0.114	*w* = 1/[σ^2^(*F*_o_^2^) + (0.0296*P*)^2^ + 1.4177*P*] where *P* = (*F*_o_^2^ + 2*F*_c_^2^)/3
*S* = 1.08	(Δ/σ)_max_ = 0.009
4989 reflections	Δρ_max_ = 0.22 e Å^−^^3^
379 parameters	Δρ_min_ = −0.20 e Å^−^^3^
0 restraints	Extinction correction: *SHELXL97* (Sheldrick, 2008), Fc^*^=kFc[1+0.001xFc^2^λ^3^/sin(2θ)]^-1/4^
Primary atom site location: structure-invariant direct methods	Extinction coefficient: 0.0025 (5)

### Special details

**Table:**

Geometry. All e.s.d.'s (except the e.s.d. in the dihedral angle between two l.s. planes) are estimated using the full covariance matrix. The cell e.s.d.'s are taken into account individually in the estimation of e.s.d.'s in distances, angles and torsion angles; correlations between e.s.d.'s in cell parameters are only used when they are defined by crystal symmetry. An approximate (isotropic) treatment of cell e.s.d.'s is used for estimating e.s.d.'s involving l.s. planes.
Refinement. Refinement of *F*^2^ against ALL reflections. The weighted *R*-factor *wR* and goodness of fit *S* are based on *F*^2^, conventional *R*-factors *R* are based on *F*, with *F* set to zero for negative *F*^2^. The threshold expression of *F*^2^ > σ(*F*^2^) is used only for calculating *R*-factors(gt) *etc*. and is not relevant to the choice of reflections for refinement. *R*-factors based on *F*^2^ are statistically about twice as large as those based on *F*, and *R*- factors based on ALL data will be even larger.

### Fractional atomic coordinates and isotropic or equivalent isotropic displacement parameters (Å^2^)

**Table:**

	*x*	*y*	*z*	*U*_iso_*/*U*_eq_	Occ. (<1)
N20	0.5777 (2)	0.96003 (16)	0.67143 (17)	0.0415 (6)	
Cl	0.37477 (6)	0.87567 (5)	0.53461 (4)	0.0384 (2)	
O1	0.52861 (16)	0.85019 (13)	0.43127 (13)	0.0406 (5)	
C1	0.5296 (2)	0.78792 (17)	0.36475 (17)	0.0305 (6)	
C2	0.4550 (2)	0.71593 (17)	0.33306 (17)	0.0306 (6)	
C3	0.4654 (2)	0.65504 (17)	0.26836 (17)	0.0305 (6)	
H3	0.4158	0.6053	0.2469	0.037*	
C4	0.5451 (2)	0.66375 (17)	0.23373 (17)	0.0299 (6)	
C5	0.6164 (2)	0.73712 (17)	0.26655 (17)	0.0339 (6)	
H5	0.6712	0.7449	0.2435	0.041*	
C6	0.6106 (2)	0.79978 (17)	0.33215 (18)	0.0329 (6)	
C7	0.3669 (2)	0.70383 (19)	0.36795 (19)	0.0400 (7)	
H7A	0.3209	0.6520	0.3355	0.060*	
H7B	0.3249	0.7614	0.3575	0.060*	
H7C	0.3975	0.6902	0.4336	0.060*	
C8	0.6908 (2)	0.8769 (2)	0.3682 (2)	0.0464 (8)	
H8A	0.7396	0.8766	0.3355	0.070*	
H8B	0.7313	0.8674	0.4337	0.070*	
H8C	0.6539	0.9371	0.3588	0.070*	
C9	0.5473 (2)	0.59489 (17)	0.15972 (17)	0.0331 (6)	
C10	0.4500 (2)	0.61622 (19)	0.07168 (17)	0.0428 (7)	
H10A	0.4526	0.6815	0.0542	0.064*	
H10B	0.3845	0.6054	0.0827	0.064*	
H10C	0.4511	0.5753	0.0223	0.064*	
C11	0.6480 (2)	0.60593 (19)	0.13774 (19)	0.0430 (7)	
H11A	0.7112	0.5997	0.1941	0.064*	
H11B	0.6478	0.6676	0.1109	0.064*	
H11C	0.6490	0.5575	0.0942	0.064*	
C12	0.5458 (2)	0.49417 (17)	0.19209 (16)	0.0271 (6)	
C13	0.6213 (2)	0.46605 (17)	0.27644 (17)	0.0289 (6)	
H13	0.6688	0.5114	0.3141	0.035*	
C14	0.62964 (19)	0.37446 (17)	0.30749 (16)	0.0275 (6)	
C15	0.5596 (2)	0.30877 (17)	0.25142 (17)	0.0289 (6)	
C16	0.4806 (2)	0.33380 (17)	0.16800 (17)	0.0284 (6)	
C17	0.4755 (2)	0.42643 (17)	0.14003 (16)	0.0276 (6)	
H17	0.4220	0.4441	0.0833	0.033*	
C18	0.7107 (2)	0.34792 (18)	0.40014 (17)	0.0355 (7)	
H18A	0.7588	0.3004	0.3927	0.053*	
H18B	0.6742	0.3229	0.4382	0.053*	
H18C	0.7519	0.4033	0.4297	0.053*	
C19	0.4016 (2)	0.26264 (18)	0.11083 (18)	0.0381 (7)	
H19A	0.3731	0.2274	0.1497	0.057*	
H19B	0.4370	0.2197	0.0831	0.057*	
H19C	0.3432	0.2945	0.0626	0.057*	
O2	0.56282 (16)	0.21611 (12)	0.27588 (14)	0.0407 (5)	
C21	0.5685 (2)	0.9709 (2)	0.7521 (2)	0.0462 (8)	
H21	0.5092	0.9447	0.7618	0.055*	
C22	0.6438 (2)	1.01925 (19)	0.8208 (2)	0.0420 (7)	
H22	0.6367	1.0258	0.8781	0.050*	
C23	0.7310 (2)	1.05919 (17)	0.80820 (18)	0.0324 (6)	
C24	0.7377 (2)	1.04480 (18)	0.72336 (19)	0.0374 (7)	
H24	0.7967	1.0691	0.7120	0.045*	
C25	0.6597 (2)	0.99569 (19)	0.65561 (19)	0.0424 (7)	
H25	0.6646	0.9873	0.5976	0.051*	
C26	0.8127 (2)	1.11584 (19)	0.88468 (19)	0.0408 (7)	
C27	0.8489 (7)	1.0589 (5)	0.9743 (5)	0.067 (3)	0.619 (7)
H27A	0.7867	1.0424	0.9885	0.100*	0.619 (7)
H27B	0.8982	1.0964	1.0243	0.100*	0.619 (7)
H27C	0.8847	1.0018	0.9670	0.100*	0.619 (7)
C28	0.7510 (4)	1.2038 (3)	0.9005 (4)	0.0549 (19)	0.619 (7)
H28A	0.7999	1.2425	0.9495	0.082*	0.619 (7)
H28B	0.6923	1.1828	0.9177	0.082*	0.619 (7)
H28C	0.7224	1.2404	0.8440	0.082*	0.619 (7)
C29	0.9038 (5)	1.1479 (5)	0.8648 (4)	0.070 (2)	0.619 (7)
H29A	0.9496	1.1870	0.9153	0.105*	0.619 (7)
H29B	0.8787	1.1843	0.8081	0.105*	0.619 (7)
H29C	0.9444	1.0938	0.8578	0.105*	0.619 (7)
C30	0.8223 (8)	1.2143 (6)	0.8482 (7)	0.069 (4)	0.381 (7)
H30A	0.8707	1.2524	0.8981	0.104*	0.381 (7)
H30B	0.7520	1.2438	0.8237	0.104*	0.381 (7)
H30C	0.8504	1.2089	0.7995	0.104*	0.381 (7)
C31	0.8037 (13)	1.1145 (11)	0.9700 (8)	0.104 (7)	0.381 (7)
H31A	0.8624	1.1501	1.0141	0.156*	0.381 (7)
H31B	0.8065	1.0498	0.9910	0.156*	0.381 (7)
H31C	0.7357	1.1426	0.9647	0.156*	0.381 (7)
C32	0.9270 (7)	1.0667 (8)	0.8952 (7)	0.073 (4)	0.381 (7)
H32A	0.9270	1.0013	0.9134	0.109*	0.381 (7)
H32B	0.9857	1.0999	0.9420	0.109*	0.381 (7)
H32C	0.9361	1.0698	0.8366	0.109*	0.381 (7)
C41	0.3686 (5)	0.8541 (4)	0.9056 (4)	0.0463 (15)	0.50
H41A	0.2966	0.8651	0.9039	0.069*	0.50
H41B	0.3958	0.7950	0.9372	0.069*	0.50
H41C	0.3667	0.8508	0.8430	0.069*	0.50
C42	0.4352 (3)	0.9272 (3)	0.9523 (3)	0.0648 (11)	
C43	0.4328 (3)	1.0136 (3)	0.9105 (3)	0.0702 (11)	
H43	0.3863	1.0227	0.8486	0.084*	
C44	0.5031 (3)	0.9144 (3)	1.0423 (3)	0.0699 (11)	
H44	0.5054	0.8559	1.0714	0.084*	
H20	0.511 (3)	0.927 (2)	0.622 (2)	0.092 (12)*	
H2	0.593 (3)	0.208 (2)	0.333 (2)	0.078 (12)*	
H1	0.477 (3)	0.842 (3)	0.453 (3)	0.099 (15)*	

### Atomic displacement parameters (Å^2^)

**Table:**

	*U*^11^	*U*^22^	*U*^33^	*U*^12^	*U*^13^	*U*^23^
N20	0.0395 (15)	0.0352 (14)	0.0404 (15)	−0.0018 (12)	0.0044 (13)	−0.0008 (11)
Cl	0.0462 (4)	0.0377 (4)	0.0290 (4)	−0.0071 (3)	0.0116 (3)	−0.0025 (3)
O1	0.0424 (12)	0.0351 (11)	0.0439 (12)	−0.0056 (9)	0.0160 (10)	−0.0110 (9)
C1	0.0322 (15)	0.0251 (14)	0.0295 (15)	0.0055 (12)	0.0064 (12)	0.0006 (12)
C2	0.0269 (14)	0.0276 (14)	0.0339 (15)	0.0018 (12)	0.0077 (12)	0.0022 (12)
C3	0.0317 (15)	0.0253 (14)	0.0302 (14)	−0.0028 (12)	0.0068 (12)	0.0008 (11)
C4	0.0343 (15)	0.0258 (14)	0.0281 (14)	0.0027 (12)	0.0103 (12)	0.0048 (11)
C5	0.0360 (16)	0.0313 (15)	0.0348 (16)	0.0003 (13)	0.0139 (13)	0.0028 (13)
C6	0.0321 (15)	0.0275 (14)	0.0342 (15)	0.0002 (12)	0.0069 (13)	0.0015 (12)
C7	0.0374 (17)	0.0352 (16)	0.0476 (18)	−0.0033 (13)	0.0163 (14)	−0.0082 (13)
C8	0.0418 (18)	0.0444 (18)	0.0527 (19)	−0.0125 (15)	0.0174 (15)	−0.0088 (15)
C9	0.0410 (16)	0.0316 (15)	0.0288 (14)	−0.0016 (13)	0.0154 (13)	−0.0004 (12)
C10	0.059 (2)	0.0350 (16)	0.0285 (15)	0.0023 (15)	0.0094 (14)	0.0037 (13)
C11	0.059 (2)	0.0370 (17)	0.0422 (17)	−0.0069 (14)	0.0301 (15)	0.0006 (13)
C12	0.0317 (14)	0.0270 (14)	0.0266 (14)	0.0015 (12)	0.0154 (12)	−0.0001 (11)
C13	0.0254 (14)	0.0324 (15)	0.0275 (14)	−0.0024 (12)	0.0085 (12)	−0.0045 (12)
C14	0.0266 (14)	0.0306 (14)	0.0251 (13)	0.0009 (12)	0.0097 (11)	−0.0002 (12)
C15	0.0320 (15)	0.0267 (14)	0.0288 (14)	−0.0008 (12)	0.0122 (12)	0.0009 (11)
C16	0.0283 (14)	0.0285 (14)	0.0285 (14)	−0.0017 (12)	0.0108 (12)	−0.0037 (11)
C17	0.0280 (14)	0.0324 (15)	0.0218 (13)	0.0024 (12)	0.0089 (11)	0.0015 (11)
C18	0.0333 (15)	0.0319 (15)	0.0341 (15)	0.0006 (12)	0.0046 (12)	0.0006 (12)
C19	0.0379 (16)	0.0365 (16)	0.0341 (16)	−0.0034 (13)	0.0069 (13)	−0.0035 (13)
O2	0.0527 (13)	0.0278 (11)	0.0323 (12)	−0.0025 (9)	0.0053 (10)	0.0012 (9)
C21	0.0412 (18)	0.0473 (19)	0.052 (2)	−0.0104 (15)	0.0204 (16)	−0.0086 (16)
C22	0.0415 (17)	0.0466 (18)	0.0400 (17)	−0.0110 (15)	0.0177 (14)	−0.0086 (14)
C23	0.0287 (15)	0.0292 (14)	0.0366 (16)	0.0022 (12)	0.0093 (13)	0.0041 (12)
C24	0.0367 (16)	0.0351 (16)	0.0416 (17)	0.0004 (13)	0.0160 (14)	0.0060 (13)
C25	0.054 (2)	0.0356 (17)	0.0353 (17)	0.0034 (15)	0.0147 (15)	0.0055 (13)
C26	0.0346 (16)	0.0393 (17)	0.0404 (17)	−0.0090 (14)	0.0051 (13)	−0.0003 (14)
C27	0.074 (6)	0.055 (4)	0.040 (4)	−0.011 (3)	−0.014 (3)	0.004 (3)
C28	0.049 (3)	0.041 (3)	0.060 (4)	−0.002 (3)	0.004 (3)	−0.016 (3)
C29	0.052 (4)	0.093 (6)	0.068 (4)	−0.038 (4)	0.025 (3)	−0.024 (4)
C30	0.062 (7)	0.044 (6)	0.073 (7)	−0.004 (5)	−0.007 (6)	−0.003 (5)
C31	0.115 (14)	0.155 (17)	0.045 (7)	−0.097 (13)	0.032 (9)	−0.043 (11)
C32	0.042 (6)	0.060 (7)	0.086 (8)	−0.008 (5)	−0.009 (5)	−0.008 (6)
C41	0.040 (4)	0.054 (4)	0.046 (4)	0.009 (3)	0.017 (3)	0.002 (3)
C42	0.047 (2)	0.058 (2)	0.105 (3)	−0.0047 (18)	0.047 (2)	−0.027 (2)
C43	0.053 (2)	0.080 (3)	0.093 (3)	0.005 (2)	0.044 (2)	−0.017 (2)
C44	0.054 (2)	0.070 (3)	0.099 (3)	0.004 (2)	0.045 (2)	−0.012 (2)

### Geometric parameters (Å, º)

**Table:**

N20—C25	1.331 (4)	C19—H19A	0.9800
N20—C21	1.335 (4)	C19—H19B	0.9800
N20—Cl	3.035 (3)	C19—H19C	0.9800
N20—H20	1.06 (4)	O2—H2	0.85 (3)
Cl—H20	1.98 (4)	C21—C22	1.365 (4)
Cl—H1	2.27 (4)	C21—H21	0.9500
O1—C1	1.382 (3)	C22—C23	1.394 (4)
O1—H1	0.90 (4)	C22—H22	0.9500
C1—C6	1.389 (4)	C23—C24	1.392 (4)
C1—C2	1.398 (3)	C23—C26	1.528 (4)
C2—C3	1.390 (3)	C24—C25	1.379 (4)
C2—C7	1.502 (4)	C24—H24	0.9500
C3—C4	1.389 (4)	C25—H25	0.9500
C3—H3	0.9500	C26—C31	1.398 (12)
C4—C5	1.389 (3)	C26—C29	1.459 (6)
C4—C9	1.538 (3)	C26—C27	1.544 (8)
C5—C6	1.395 (4)	C26—C30	1.547 (9)
C5—H5	0.9500	C26—C28	1.584 (6)
C6—C8	1.504 (3)	C26—C32	1.653 (10)
C7—H7A	0.9800	C27—H27A	0.9800
C7—H7B	0.9800	C27—H27B	0.9800
C7—H7C	0.9800	C27—H27C	0.9800
C8—H8A	0.9800	C28—H28A	0.9800
C8—H8B	0.9800	C28—H28B	0.9800
C8—H8C	0.9800	C28—H28C	0.9800
C9—C12	1.533 (3)	C29—H29A	0.9800
C9—C11	1.539 (4)	C29—H29B	0.9800
C9—C10	1.543 (4)	C29—H29C	0.9800
C10—H10A	0.9800	C30—H30A	0.9800
C10—H10B	0.9800	C30—H30B	0.9800
C10—H10C	0.9800	C30—H30C	0.9800
C11—H11A	0.9800	C31—H31A	0.9800
C11—H11B	0.9800	C31—H31B	0.9800
C11—H11C	0.9800	C31—H31C	0.9800
C12—C17	1.391 (3)	C32—H32A	0.9800
C12—C13	1.398 (3)	C32—H32B	0.9800
C13—C14	1.390 (3)	C32—H32C	0.9800
C13—H13	0.9500	C41—C42	1.398 (6)
C14—C15	1.391 (3)	C41—H41A	0.9800
C14—C18	1.510 (3)	C41—H41B	0.9800
C15—O2	1.378 (3)	C41—H41C	0.9800
C15—C16	1.395 (3)	C42—C44	1.383 (5)
C16—C17	1.392 (3)	C42—C43	1.398 (5)
C16—C19	1.507 (3)	C43—C44^i^	1.373 (5)
C17—H17	0.9500	C43—H43	0.9500
C18—H18A	0.9800	C44—C43^i^	1.373 (5)
C18—H18B	0.9800	C44—H44	0.9500
C18—H18C	0.9800		
			
C25—N20—C21	121.4 (3)	C16—C19—H19C	109.5
C25—N20—Cl	128.1 (2)	H19A—C19—H19C	109.5
C21—N20—Cl	109.82 (19)	H19B—C19—H19C	109.5
C25—N20—H20	125 (2)	C15—O2—H2	113 (2)
C21—N20—H20	113 (2)	N20—C21—C22	120.4 (3)
H20—Cl—H1	82.2 (14)	N20—C21—H21	119.8
C1—O1—H1	116 (2)	C22—C21—H21	119.8
O1—C1—C6	116.0 (2)	C21—C22—C23	121.0 (3)
O1—C1—C2	122.3 (2)	C21—C22—H22	119.5
C6—C1—C2	121.6 (2)	C23—C22—H22	119.5
C3—C2—C1	117.7 (2)	C24—C23—C22	116.4 (2)
C3—C2—C7	120.8 (2)	C24—C23—C26	123.2 (2)
C1—C2—C7	121.5 (2)	C22—C23—C26	120.5 (2)
C4—C3—C2	122.9 (2)	C25—C24—C23	120.8 (3)
C4—C3—H3	118.6	C25—C24—H24	119.6
C2—C3—H3	118.6	C23—C24—H24	119.6
C5—C4—C3	117.2 (2)	N20—C25—C24	120.0 (3)
C5—C4—C9	123.9 (2)	N20—C25—H25	120.0
C3—C4—C9	118.9 (2)	C24—C25—H25	120.0
C4—C5—C6	122.5 (3)	C31—C26—C29	126.9 (6)
C4—C5—H5	118.8	C31—C26—C23	116.9 (5)
C6—C5—H5	118.8	C29—C26—C23	114.7 (3)
C1—C6—C5	118.1 (2)	C29—C26—C27	111.2 (5)
C1—C6—C8	120.7 (2)	C23—C26—C27	109.2 (3)
C5—C6—C8	121.2 (3)	C31—C26—C30	115.0 (8)
C2—C7—H7A	109.5	C29—C26—C30	55.7 (5)
C2—C7—H7B	109.5	C23—C26—C30	109.1 (4)
H7A—C7—H7B	109.5	C27—C26—C30	141.3 (5)
C2—C7—H7C	109.5	C31—C26—C28	67.4 (8)
H7A—C7—H7C	109.5	C29—C26—C28	109.0 (4)
H7B—C7—H7C	109.5	C23—C26—C28	106.1 (3)
C6—C8—H8A	109.5	C27—C26—C28	106.2 (4)
C6—C8—H8B	109.5	C30—C26—C28	57.2 (5)
H8A—C8—H8B	109.5	C31—C26—C32	108.5 (8)
C6—C8—H8C	109.5	C29—C26—C32	46.9 (4)
H8A—C8—H8C	109.5	C23—C26—C32	103.1 (4)
H8B—C8—H8C	109.5	C27—C26—C32	73.8 (5)
C12—C9—C4	110.1 (2)	C30—C26—C32	102.6 (6)
C12—C9—C11	107.2 (2)	C28—C26—C32	148.7 (4)
C4—C9—C11	112.3 (2)	C26—C27—H27A	109.5
C12—C9—C10	111.8 (2)	C26—C27—H27B	109.5
C4—C9—C10	107.6 (2)	C26—C27—H27C	109.5
C11—C9—C10	107.9 (2)	C26—C28—H28A	109.5
C9—C10—H10A	109.5	C26—C28—H28B	109.5
C9—C10—H10B	109.5	C26—C28—H28C	109.5
H10A—C10—H10B	109.5	C26—C29—H29A	109.5
C9—C10—H10C	109.5	C26—C29—H29B	109.5
H10A—C10—H10C	109.5	C26—C29—H29C	109.5
H10B—C10—H10C	109.5	C26—C30—H30A	109.5
C9—C11—H11A	109.5	C26—C30—H30B	109.5
C9—C11—H11B	109.5	H30A—C30—H30B	109.5
H11A—C11—H11B	109.5	C26—C30—H30C	109.5
C9—C11—H11C	109.5	H30A—C30—H30C	109.5
H11A—C11—H11C	109.5	H30B—C30—H30C	109.5
H11B—C11—H11C	109.5	C26—C31—H31A	109.5
C17—C12—C13	117.0 (2)	C26—C31—H31B	109.5
C17—C12—C9	123.5 (2)	H31A—C31—H31B	109.5
C13—C12—C9	119.4 (2)	C26—C31—H31C	109.5
C14—C13—C12	122.7 (2)	H31A—C31—H31C	109.5
C14—C13—H13	118.6	H31B—C31—H31C	109.5
C12—C13—H13	118.6	C26—C32—H32A	109.5
C13—C14—C15	117.9 (2)	C26—C32—H32B	109.5
C13—C14—C18	120.8 (2)	H32A—C32—H32B	109.5
C15—C14—C18	121.3 (2)	C26—C32—H32C	109.5
O2—C15—C14	122.1 (2)	H32A—C32—H32C	109.5
O2—C15—C16	116.3 (2)	H32B—C32—H32C	109.5
C14—C15—C16	121.6 (2)	C42—C41—H41A	109.5
C17—C16—C15	118.2 (2)	C42—C41—H41B	109.5
C17—C16—C19	121.0 (2)	H41A—C41—H41B	109.5
C15—C16—C19	120.7 (2)	C42—C41—H41C	109.5
C12—C17—C16	122.4 (2)	H41A—C41—H41C	109.5
C12—C17—H17	118.8	H41B—C41—H41C	109.5
C16—C17—H17	118.8	C44—C42—C41	119.3 (4)
C14—C18—H18A	109.5	C44—C42—C43	119.4 (4)
C14—C18—H18B	109.5	C41—C42—C43	121.3 (5)
H18A—C18—H18B	109.5	C44^i^—C43—C42	120.8 (4)
C14—C18—H18C	109.5	C44^i^—C43—H43	119.6
H18A—C18—H18C	109.5	C42—C43—H43	119.6
H18B—C18—H18C	109.5	C43^i^—C44—C42	119.9 (4)
C16—C19—H19A	109.5	C43^i^—C44—H44	120.1
C16—C19—H19B	109.5	C42—C44—H44	120.1
H19A—C19—H19B	109.5		

Symmetry code: (i) −*x*+1, −*y*+2, −*z*+2.

### Hydrogen-bond geometry (Å, º)

**Table:**

*D*—H···*A*	*D*—H	H···*A*	*D*···*A*	*D*—H···*A*
N20—H20···Cl	1.06 (4)	1.98 (4)	3.035 (3)	173 (3)
O2—H2···Cl^ii^	0.85 (3)	2.30 (3)	3.079 (2)	153 (3)
O1—H1···Cl	0.90 (4)	2.27 (4)	3.118 (2)	157 (3)

Symmetry code: (ii) −*x*+1, −*y*+1, −*z*+1.
